# Fostering effective and sustainable scientific collaboration and knowledge exchange: a workshop-based approach to establish a national ecological observatory network (NEON) domain-specific user group

**DOI:** 10.1007/s00484-024-02673-x

**Published:** 2024-04-24

**Authors:** Alison Donnelly, Ankur R. Desai, Katherine A. Heckman, Lucas E. Nave, Michael J. Cramer, Marie Faust, Peter Weishampel, Caleb Slemmons, Christian G. Andresen, Edward Ayres, Stacy R. Cotey, Kathryn M. Docherty, Joshua Hatzis, Kathryn Hofmeister, Jalene M. LaMontagne, John D. Lenters, Noah R. Lottig, Amy M. Marcarelli, Jessica Miesel, Jason Riddle, Meghan Salmon-Tumas, Mike D. SanClements, Subash Sapkota, Mark D. Schwartz, Puja Sharma, Ojaswee Shrestha, Geoffrey Vincent, Angela Waupochick, Ting Zheng, Zhiwei Ye

**Affiliations:** 1 Department of Geography, University of Wisconsin-Milwaukee, WI, USA; 2 Department of Atmospheric and Oceanic Sciences, University of Wisconsin-Madison, Milwaukee, WI USA; 3 USDA Forest Service, Houghton, MI USA; 4 College of Forest Resources and Environmental Science, Michigan Technological University, Houghton, MI USA; 5 Environmental Research Center and Department of Biological Sciences, University of Notre Dame, Notre Dame, IN USA; 6 National Ecological Observatory Network, Battelle, Boulder, CO USA; 7 Department of Geography, University of Wisconsin-Madison, Madison, WI USA; 8 Department of Biological Sciences, Western Michigan University, Michigan, USA; 9 Environmental Studies Program, University of Wisconsin-Oshkosh, Oshkosh, WI USA; 10 Department of Biological Sciences, DePaul University, Chicago, IL USA; 11 University of Michigan Biological Station, University of Michigan, Pellston, MI USA; 12 Center for Limnology, University of Wisconsin-Madison, Madison, WI USA; 13 Department of Biological Sciences, Michigan Technological University, Houghton, MI USA; 14 Department of Plant, Soil and Microbial Sciences, Michigan State University, Michigan, USA; 15 College of Natural Resources, University of Wisconsin-Stevens Point, Stevens Point, WI USA; 16 Department of Environmental Sciences, Northland College, Ashland, WI USA; 17 University of Louisiana Monroe, Louisiana Monroe, LA USA; 18 Department of Forest and Wildlife Ecology, University of Wisconsin-Madison, Madison, WI USA

**Keywords:** NEON great lakes user group, Workshop framework, Researcher engagement

## Abstract

The decision to establish a network of researchers centers on identifying shared research goals. Ecologically specific regions, such as the USA’s National Ecological Observatory Network’s (NEON’s) eco-climatic domains, are ideal locations by which to assemble researchers with a diverse range of expertise but focused on the same set of ecological challenges. The recently established Great Lakes User Group (GLUG) is NEON’s first domain specific ensemble of researchers, whose goal is to address scientific and technical issues specific to the Great Lakes Domain 5 (D05) by using NEON data to enable advancement of ecosystem science. Here, we report on GLUG’s kick off workshop, which comprised lightning talks, keynote presentations, breakout brainstorming sessions and field site visits. Together, these activities created an environment to foster and strengthen GLUG and NEON user engagement. The tangible outcomes of the workshop exceeded initial expectations and include plans for (i) two journal articles (in addition to this one), (ii) two potential funding proposals, (iii) an assignable assets request and (iv) development of classroom activities using NEON datasets. The success of this 2.5-day event was due to a combination of factors, including establishment of clear objectives, adopting engaging activities and providing opportunities for active participation and inclusive collaboration with diverse participants. Given the success of this approach we encourage others, wanting to organize similar groups of researchers, to adopt the workshop framework presented here which will strengthen existing collaborations and foster new ones, together with raising greater awareness and promotion of use of NEON datasets. Establishing domain specific user groups will help bridge the scale gap between site level data collection and addressing regional and larger ecological challenges.

**Supplementary Information:**

The online version contains supplementary material available at 10.1007/s00484-024-02673-x.

## Introduction

Workshops have been successfully used, in the past, in many contexts, such as, to problem solve, innovate, brainstorm, build community, etc. (Donnelly et al., 2006; Ørngreen and Levinsen 2017). With effective organization and well-defined goals, workshops become valuable and productive once a motivated team is assembled, often leading to unexpected outcomes that yield new knowledge and inspire novel insights (Ørngreen and Levinsen 2017). When it comes to advancing ecology, and specifically concepts in the field of macrosystems ecology, researchers across multiple disciplines need close cooperation (Nagy et al. 2021) regardless of geographical scale or location.

The National Ecological Observatory Network (NEON) is a US ecological research infrastructure and observatory intended to provide spatially distributed biotic and abiotic ecologically relevant observations over a long time period (~ 3 decades). NEON is one of the emerging networks of Global Ecosystem Research Infrastructures (GERI) that have arisen in the past decades worldwide and all of which have established various mechanisms for scientist and community interaction, interoperability, and advancing global ecosystem research (Loescher et al. 2022). NEON’s specific aim is to establish a baseline for developing and testing theories linked to Grand Challenges in ecology related to biodiversity, disease spread, invasive species, land use, and climate change impacts. The focus is on a continental scale, specifically within the United States (Heffernan et al., 2014). The objectives align with similar initiatives such as Australia’s Terrestrial Ecosystem Research Network (TERN) or the South Africa Environmental Observation Network (SAEON). NEON’s research infrastructure is replicated across a set of ecoclimatically grouped domains, one of which is D05, encompassing terrestrial and aquatic ecosystems in US states adjacent to the Laurentian Great Lakes. The specific contribution to the Grand Challenges identified in the NOEN Great Lakes Domain (D05) are, (i) understanding how forest and peatland carbon fluxes and greenhouse gas dynamics respond to climate change, (ii) monitoring range expansion of vector-spread diseases such as Lyme and other tick-borne diseases including those spread by mosquito, and (iii) tracking the impact of terrestrial and aquatic invasive species on habitat structure, biogeochemistry and response to climate change. Achieving these goals requires multiple modes of engagement of ecologists, including workshops and reimagined training (Farrell et al., 2021; Nagy et al. 2021).

While in principle, network building may occur organically, in practice, this approach needs to be facilitated through the bottom-up and building from existing networks of researchers, experimental sites, and stakeholders that exist within distinct geographic regions. We argue that physical proximity helps foster researcher relationships and encourage and enable cross-disciplinary research. Within NEONs 20 ecoclimatic Domains are both local staff (Schimel 2011; Thorpe et al. 2016) and long-standing research networks. Therefore, leveraging both groups together with their resources helps strengthen existing relationships and collaborations (SanClements et al. 2022).

The long-term value of NEON or other research infrastructures with similar top-down approaches to management depends on regional organization by knowledgeable people engaged in addressing ecological challenges at granular scales from which macroscale experiments and collaborative research projects will originate. Here we report the outcomes of a workshop designed to launch a newly (2022) formed Domain-specific user group with diverse research interests and outline how other domains might replicate the most successful elements of our experience. Prior to the workshop, a stakeholder working group had been established in D05, i.e. the Great Lakes User Group or GLUG for short. While a series of online meetings helped establish mutual objectives and goals, the need for an in-person workshop to advance our mission became evident.

### Workshop need, format and organization

#### Establish need, set goals and seek workshop funding

Using an already established NEON database of researchers and publications coupled with contacts from NEON Technical Working Groups, the NEON Outreach Specialist identified a number of researchers in the Great Lakes Domain (D05). Subsequently, a survey was circulated among the list of 20 researchers to determine the need and willingness to form a domain specific user group. A virtual meeting was held in January 2022 which attracted 21 attendees comprising 15 institutions. Periodic recurring virtual meetings were subsequently held, leading to the drafting of a charter, outlining objectives, scope, approach and membership of the GLUG (https://www.neonscience.org/neon-great-lakes-user-group).

The primary goal for the workshop was to coordinate researchers in the Great Lakes region to identify and integrate biotic and abiotic ecologically focused data from different sources to identify and advance understanding of specific challenges within the Great Lakes domain. The hope was that success and lessons learned from this model could provide a template for other NEON domains, or similar entities, to develop their own user groups. In order to ensure the workshop was sufficiently resourced and could attract participants from diverse backgrounds who would not be prevented from attending due to a lack of funding we inquired and received support from the National Science Foundation (NSF) under the category ‘Conference grants support’.

#### Workshop planning

The organizing committee felt that, compared to an online or hybrid approach, an in-person workshop would foster valuable networking opportunities, provide a collaborative atmosphere and maximize researcher exposure to the field activities conducted by science staff at NEON sites. The particular venue (Kemp Natural Resources Station, operated by UW-Madison) was chosen based on the suitability and co-location of lodging, seminar rooms, meal preparation facilities, and social space amenities. A semi-flexible schedule was adopted to allow participants to move between breakout sessions, foster engagement and enhance motivation. This approach has been suggested to reduce fatigue due to information overload, accommodate unexpected events and enhance knowledge retention. With that in mind, the workshop was designed around brief presentations, site visits, keynote presentations and breakout sessions, punctuated by coffee breaks, lunch and dinner for informal networking with a final session on future steps and maintaining momentum (Table 1). After deliberation, we opted to invite two guest speakers to make 15 min presentations each, one on networked research with NEON data, and the other on use of NEON data in education.

**Table 1:** Format and structure of the Great Lakes User Group kick-off workshop at Kemp Natural Resources Station, Woodruff, Wisconsin September 2023

Time	Day 1	Day 2	Day 3
Early morning	Registration	Keynote speakers (15 min each) and discussion	Breakout groups
	Welcome address		
	Aims and structure of GLUG and the workshop		Report from breakouts
		Breakout groups	Reflections, next steps and wrap-up
**Coffee break**			
Mid-morning	Lightening presentations from 10 participants	Breakout groups	Organizing committee meet to synthesize workshop and plan next steps
	Breakout session pitches		
	Breakout groups begin		
**Lunch break**			
Early afternoon	Report from breakouts	Report from breakouts	
Mid– late afternoon	Field site visit: Aquatic site, Little Rock	Field site visit: Terrestrial site, Treehaven	
Evening	Dinner	Dinner	

Dinner was provided the evening before the workshop started. A detailed schedule is available in Table S1

#### Workshop participants, venue and format

Over the course of the 2.5-days, 28 participants joined the workshop for some or all of the sessions and represented a wide range of career stages, including undergraduate students. The majority (16) of participants were in the mid-career stage, with representation from government agencies, research universities and 4-year colleges focusing on teaching. Researchers from five different States, including Illinois (1), Indiana (1), Louisiana (1), Michigan (8) and Wisconsin (12) attended, and NEON (4) personnel were also represented. The group embodied a broad range of both research expertise and technical skills covering areas such as wildlife biology, population ecology, disease ecology, aquatic chemistry, vegetation dynamics and phenology, remote sensing, and soil structure and function.

The venue provided ample room, resources and facilities to accommodate an effective workshop. Breakfast, lunch, snacks and one of the dinners (sample shopping list available in supplemental materials Table S2) were prepared by the organizing committee with help from participants. Proximity to two NEON sites, one aquatic and one terrestrial, resulted in minimal travel time to and from the field facilities.

#### Breakout sessions: design, topic selection and effectiveness

The breakout sessions were designed to deliver the outcomes of using NEON data to draft a research proposal, scientific paper(s) or other products, related to specific ecological challenges facing the Great Lakes eco-climatic domain. The participation of NEON staff was particularly beneficial in developing the breakout session products and ideas. One drawback of this approach is that the topics identified were limited to the expertise in the room. A breakout session coordinator was selected whose role was to solicit topics from the group, summarize the key points of the topic, seek participants to work on the topic and facilitate regular reporting of each breakout group to the entire delegation. After group discussion, five of seven proposed topics moved forward (Table 2).

**Table 2:** List of proposed breakout session topics and products

Proposed breakout session	Potential product
Developing the use of National Ecological Observatory Network data in a classroom setting.	Classroom activity.
How disturbance gradients affect biogeochemistry, carbon cycling, insects, mammals, disease, invasive species, biophysical parameters, etc.	Full research proposal.
Seasonal changes in water and populations that occur at National Ecological Observatory Network sites.	Scientific paper(s).
The effects of climate change on the Great Lakes Domain.	
Integrating aquatic and terrestrial data.	Small research proposal.
How representative are the sites of the entire eco-climatic domain?	
Use of extreme events, such as the upcoming El Niño, to make inferences about climate change.	Scientific paper(s).

Sessions with ‘strikethrough’ were rolled into other sessions

Each of the breakout groups appointed a lead and a rapporteur to manage the group and document the discussion. Pre-populated questions were used to kick-start the discussion. These included: (i) are there regionally-specific (D05, Great Lakes) questions that can be addressed with existing NEON data availability?, (ii) what is the most pressing scientific question in your field at the moment?, and (iii) what instrumentation and/or data resources do you need to answer that question? Outcomes included ideas for papers, proposals, or NEON assignable asset requests.

## Exit survey results summary

A short exit survey (Table S3) was distributed among workshop participants by email after the workshop ended to assess strengths, weaknesses, and potential improvements. A total of 18 out of 28 participants provided responses to the survey questions with equal numbers identifying as men and women with an age range from 18 to > 65 and a majority within the 35–44 age category. Most people learned about the workshop from colleagues or were already involved with GLUG. The aspects of the workshop that respondents identified as ‘went well’ fell into three broad categories: agenda, networking opportunities and venue. The breakout groups and site tours were highlighted as providing excellent opportunities to establish new working relationships with researchers from different disciplines and to build a platform for collaboration fostering innovative ideas. It was noted that the workshop facilitated 4-year colleges to participate in research proposals and manuscript writing which otherwise may not have been possible. Overall, the majority of delegates found the workshop to be an appropriate length, with an excellent mix of activities and an ideal group size.

There were fewer responses (13) to a question relating to aspects of the workshop that required improvement. However, greater diversity among delegates, excluding weekend days and considering a different approach to the breakout sessions were identified as aspects that should be considered in future. Downsides to the breakout sessions were identified and included, the inability to easily move between sessions as they ran concurrently. A proposed solution suggested to circulate predefined topics before the workshop to encourage delegates to develop ideas prior to the breakout sessions. All respondents were keen to continue to engage with GLUG either through annual in-person or virtual meetings or for more regular meetings to develop regional activities, and all indicated that they would attend future events.

### Recommendations

A list of recommendations, based on the experience we gained from running this workshop is presented in Table 3 and should be kept in mind when developing a similar event.

**Table 3:** List of recommendations to guide the development of a user group workshop

**Logistics**
Start planning early. Ensure representation of underrepresented communities are part of the organizing committee.
Apply for funding from local (host institution), regional, or national sources, including the National Science Foundation (NSF).
Choose a venue with co-located meeting rooms, kitchen facilities and accommodation. Consider venues close to underrepresented community. A field station location is ideal.
**Workshop format**
Maintain a flexible agenda to accommodate unexpected events.
Facilitate participant introductions to enable discovery of common interests and ideas for breakout sessions.
Include diverse activities to keep participants engaged, including short presentations, breakout groups with regular reporting to the entire group, field trips and guest speakers.
Encourage participants to move between breakout sessions to share expertise and encourage interdisciplinary collaboration.
**Maintain engagement**
Write a manuscript including to include all participants and use an exit survey to gather opinions and feedback which will act as participants’ contribution to the shared manuscript.
Develop a communication strategy covering short, medium, and long-term goals, including a feedback session 2–3 weeks following the workshop, bi-monthly remote meetings, conference addons and biannual workshops.
Establish communication channels between the group and other regional, national and international networks to promote scientific collaboration and knowledge exchange within the geographic region.

### Effectiveness of the workshop and lessons learned

#### Lessons learned

The survey results and list of outcomes demonstrate that the bottom-up approach, adopted during the establishment of GLUG, succeeded in establishing an interdisciplinary ensemble of researchers providing a range of perspectives capable of tackling current and emerging complex ecological issues in the Great Lakes Domain. The success of this workshop should motivate researchers in other domains and under other similar circumstances in other geographic areas to adopt a similar approach to build a regional network of collaborators to address location specific challenges.

While workshops can be an effective means of organizing, they also have their limitations. For example, the short duration (several hours on a topic) may be insufficient to fully explore complex challenges or engage in extensive discussions. While every effort was made to include as many stakeholders as possible the selection process may have led to gaps in representation: some potential participants were unable to attend due to the geographical location of the workshop.

Despite our efforts to include under-represented minorities in the workshop we did not achieve our goal. Even though we reached out to minority-serving institutions, tribal colleges, and personal contacts, and had sufficient funds to cover expenses, we did not succeed in attracting adequate minority representation. The equal gender distribution was a positive sign of inclusivity but greater diversity in terms of career stage and under-represented groups should be a priority focus for future workshops. Proposed ways to achieve greater diversity were discussed and included, hosting a future event closer to populations of under-represented groups. However, it was acknowledged that finding a suitable venue close to field sites may be challenging. It was interesting to note that insufficient funding did not limit minority inclusion as sufficient budget was available to cover travel and other expenses yet still, funding alone was not sufficient to ensure diverse representation. Rather, setting targets for inclusion of under-represented and minority group involvement coupled with ensuring participation of these groups in the initial stages of organizing is critical. Ensuring diversity is not trivial and requires a concerted effort to include representation from multiple groups from the initial planning stage.

Despite the existence of a number of already established national and international networks of researchers within the Great Lakes region focusing, at least in part, on ecological challenges many focus primarily on one specific goal. For example, the bi-national Great Lakes Invasives Network (https://greatlakesinvasives.org/portal/index.php) focus specifically on invasive plant and animal species, while the Global Lake Ecological Observatory Network (international), the North Temperate Lakes Long-Term Ecological Research site (regional), and the Real-time Aquatic Ecosystem Observation Network (regional) focus mainly on inland freshwater issues, and Ameriflux (North American) focuses on terrestrial ecosystem carbon and water fluxes (Novick et al. 2018). The GLUG operates on a smaller scale but takes a more holistic approach incorporating a range of co-located ecologically related datasets. In order to ensure that GLUG remains relevant and complementary to these existing research networks our aim is to establish communication links with these groups to foster scientific collaboration and knowledge exchange which will be mutually beneficial.

Site visits were a particular strength of the in-person format. By visiting two NEON field sites (aquatic and terrestrial), participants were able to experience firsthand the representative conditions and spatial variability present at the sites, investigate sampling infrastructure in detail, observe data collection activities, and interact with NEON staff in the field. The value for participants of making firsthand observations and asking spontaneous questions of NEON staff in the field cannot be overstated from both inquiry and hypothesis-generation perspectives. Participants also valued the opportunity to learn about and observe work conducted under the Assignable Assets program, the formal process by which NEON reviews, approves, and is supported to conduct research activities for external investigators. Time spent in transit to and from field sites, and while preparing and enjoying meals as a group provided for shared experiences that increased the sense of belonging among participants.

The breakout sessions were identified in the exit surveys as one of the most valuable aspects of the workshop as they focused participant efforts around a common theme and channeled ideas into tangible products. However, having parallel sessions led to some frustration. Even though delegates were encouraged to move between break-out sessions in practice this was sometimes challenging especially if a delegate was not an expert of the topic under consideration and/or could not grasp the key elements of the conversation quickly. A proposed solution to this issue was a suggestion to brainstorm a list of topics during a series of online meetings before the workshop and encourage delegates to develop ideas prior to commencement of the workshop. However, the group considered that this may inhibit any creativity and innovative ideas that may emerge organically during the initial discussion of topics and themes. In fact, during the early stages of the workshop one of the delegates had an impromptu idea of exploring the impact of the upcoming El Niño event on D05. This may not have happened if topics were predefined. Identifying the appropriate number and duration of break-out sessions and the topics to be discussed, will be specific to the workshop in question and requires careful consideration during the planning phase.

#### Workshop synthesis, next steps and maintaining momentum after the workshop ends

An important component of effective workshops is that the organizers gather to synthesize the outcomes, reflect on its effectiveness, and strategize how to maintain the collaborative spirit established during the event. Analysis of the results from the exit survey provided an opportunity to share feedback with participants in the weeks following the workshop. In addition, it was decided that breakout sessions would meet regularly and present any progress to the larger GLUG at a bi-monthly meeting to be held remotely and organized by NEON. Other meeting opportunities proposed included hosting a half-day meeting at local, regional (Great Lakes Chapter of the Ecological Society of America; Science in the North Woods) or national conferences (American Geophysical Union; Ecological Society of America) that participants would already be attending. Finally, an annual workshop was also considered but the group felt that would be too frequent so it was decided to plan for future workshops as needed.

The tangible outcomes resulting from the workshop clearly demonstrates how valuable such a gathering can be, not only for the participants, but for the funding agency, those who could not attend the workshop and others thinking of hosting a similar event. Overall, the workshop provided a motivating and supportive environment for participants who clearly felt a sense of inclusion and a valuable part of the process. However, maintaining this momentum and harnessing this energy when people return to their daily work lives is challenging. Hosting regular meetings, setting clear tasks with reasonable deadlines for completion, and specific tangible outputs, such as collaborative papers, grant proposals, etc. should help maintain participant interest and motivation going forward. However, it remains to be seen how long the ‘fever’ will last and how to find innovative and interesting tasks to make the GLUG sustainable and relevant in future.

### Electronic supplementary material

Below is the link to the electronic supplementary material.

Supplementary Material 1
