# Supplementary material for: Fostering effective and sustainable scientific collaboration and knowledge exchange: a workshop-based approach to establish a national ecological observatory network (NEON) domain-specific user group
Source: Int J Biometeorol. 2024 Apr 24;68(7):1475–81. doi: 10.1007/s00484-024-02673-x (PMC11272799; doi:10.1007/s00484-024-02673-x)
Supplement: Supplementary file 1 — Supplementary Material 1 [file 484_2024_2673_MOESM1_ESM.docx]

Fostering effective and sustainable scientific collaboration and knowledge exchange: A workshop-based approach to establish a National Ecological Observatory Network (NEON) Domain-specific user group

Donnelly A^1*^, Desai AR^2^, Heckman K^3^, Nave LE^4^, Cramer MJ^5^, Faust M^6^, Weishampel P^6^, Slemmons C^6^, Andresen C^7^, Ayres E^6^, Cotey S^4^, Docherty KM^8^, Hatzis J^1^, Hofmeister K^9^, LaMontagne JM^10^, Lenters J^11^, Lottig N^2^, Marcarelli AM^12^, Miesel J^13^, Riddle J^14^, Salmon-Tumas M^15^, SanClements MD^6^, Sapkota S^16^, Schwartz MD^1^, Sharma P^5^, Shrestha O^17^, Vincent G^15^, Waupochick A^17^, Zheng T^17^, Zhiwei Y^17^

^1^Department of Geography, University of Wisconsin-Milwaukee, Wisconsin, USA

^2^Department of Atmospheric and Oceanic Sciences, University of Wisconsin-Madison, Wisconsin, USA

^3^USDA Forest Service, USA

^4^College of Forest Resources and Environmental Science, Michigan Technological University, Houghton, Michigan, USA

^5^University of Notre Dame Environmental Research Center and Department of Biological Sciences, University of Notre Dame, Indiana, USA

^6^Battelle, National Ecological Observatory Network, Boulder, Colorado, USA

^7^Department of Geography, University of Wisconsin-Madison, Wisconsin, USA

^8^Department of Biological Sciences, Western Michigan University, Michigan, USA

^9^Environmental Studies Program, University of Wisconsin-Oshkosh, Wisconsin, USA

^10^Department of Biological Sciences, DePaul University, Chicago, Illinois, USA

^11^University of Michigan Biological Station, University of Michigan, Pellston, Michigan, USA

^12^Department of Biological Sciences, Michigan Technological University, Houghton, Michigan, USA

^13^Department of Plant, Soil and Microbial Sciences, Michigan State University, Michigan, USA

^14^College of Natural Resources, University of Wisconsin-Stevens Point, Wisconsin, USA

^15^Department of Environmental Sciences, Northland College, Ashland, Wisconsin, USA

^16^University of Louisiana Monroe, Louisiana, USA

^17^Department of Forest and Wildlife Ecology, University of Wisconsin-Madison, Wisconsin, USA

Corresponding author: Alison Donnelly ([alison.c.donnelly@gmail.com](about:blank))

**Supporting information**

**NEON’s Great Lakes User Group (GLUG) workshop Agenda**

**28-30 September 2023**

**Wednesday 27 September pre-workshop dinner**

6:00pm – 8:00pm Early registration and pre-workshop dinner

- Location: Kemp Natural Resources Station; Mead Residence Hall

**Day 1: Thursday 28 September (9am – 5pm)**

8:30 – 9:30 Breakfast and registration

- Location: Conor Forestry Center at Kemp

9:30 – 9:50 Welcome address and introductions

- Including introduction from NEON

9:50 – 10:00 Aims and structure of GLUG and the workshop

- Who we are and what we want to achieve
- Format, objectives and expected outcomes of the workshop

10:00 – 10:20 Coffee break

10:20 – 11:20 Presentations of data sets and research. Chair: Ankur

Participants will give short presentations of their data, research ideas and vision for using great lakes NEON data. During this session participants will be asked to consider specific challenges related to the Great Lakes region and to think of potential topics for breakout sessions.

| **Time** | **Presenter** | **Title** |
| --- | --- | --- |
| **Vegetation** | | |
| 10:20-10:25 | Subash Sapkota | Impact of Environmental Gradients on Seedling Distribution in a Northwoods Forest Landscape |
| 10:25-10:30 | Alison Donnelly | Detecting plant invasion across NEON eco-climatic domains |
| 10:30-10:35 | Jalene LaMontagne | Dynamics of tree reproduction, seed consumers, and ticks at NEON sites |
| 10:35-10:40 | Ting Zheng | Variations in canopy physical and functional traits drive the variations of the gross primary productivity of the Chequamegon-Nicolet National forest in northern Wisconsin |
| 10:40-10:45 | Ankur Desai | Drivers of decadal carbon fluxes across temperate ecosystems |
| 10:45-10:50 | Zhiwei Ye | The potential for mapping Leaf area index using imaging spectroscopy over Northern Wisconsin |
| **Soil** | | |
| 10:50-10:55 | Katherine Heckman | A brief tour of the soils of Domain 05 |
| 10:55-11:00 | Kathryn Docherty | How do prairie restoration and management choices influence soil carbon storage, microbial communities and spatial heterogeneity? |
| 11:00-11:05 | Luke Nave | Landscape variation in soil carbon stocks at Great Lakes NEON sites |
| **Aquatic** | | |
| 11:05-11:15 | Amy Marcarelli | Quantifying N2 flux in streams: empirical and modeling studies in NEON streams |

11:20 – 12:00 Breakout session pitches. Facilitator: Kate

Participants will give 2-3 minute impromptu ideas for Breakout sessions.

12:00 – 1:00 Breakout working group time

Participants will choose a Breakout Session to attend. Discuss research ideas focused on the Great Lakes region, potential papers and proposals. A lead and rapporteur should be chosen by consensus and the topic settled upon. Each group will report their lead, rapporteur and topic in the next session.

1:00 – 2:00 Lunch

2:00 – 2:30 Breakout groups report back to the whole group. Facilitator: Kate

Each group reports their lead, rapporteur and topic.

2:30 – 6:00 Field visit: Aquatic site LIRO

- Lake sampling, water chemistry, fish, laboratory visit, etc.

6:30 – 8:00 Dinner

- Location: Conor Forestry Center at Kemp

**Day 2: Friday 29 September (9am – 5pm)**

8:30 – 9:00 Breakfast and registration

- Location: Conor Forestry Center at Kemp

9:00 – 9:30 Guest speakers

- Network of networks. Mike SanClements, Research Initiatives Lead, NEON, Battelle.
- Small mammal data collection use in classroom. Stacy Cotey, Assistant Teaching Professor, Michigan Technological University.

9:30 – 10:15 Breakout working group time

Participants will refine their topic, discuss how to proceed, determine data sets they need, collaborators, other resources?

Leads will request potential collaborators/expertise/other.

10:15 – 10:30 Coffee break

10:30 – 12:30 Breakout working group time

Participants will discuss ideas for a potential paper, proposal (full or section), other. Assign roles and responsibilities. Participants will continue to work on their topic and develop a strategy for working together after the workshop.

12:30 – 1:30 Lunch

1:30 – 2:00 Breakout working groups report ideas. Facilitator: Kate

Each breakout group will report their ideas and vision to the whole group.

2:00 – 6:30 Field visit: Terrestrial site Treehaven

- flux tower(s), distributed plots, soil, biodiversity, phenology, mammals, etc.

7:00 – 8:30 Dinner

- Location: Minocqua Pizza Company

**Day 3: Saturday 30 September (9am – 1pm)**

9:00 – 9:30 Breakout working group time

Report back and insights

9:30 – 10:00 Reflections from organizers

- Next steps
- Sustaining the GLUG network
- Wrap up

10:00 – 10:15 Coffee break

10:15 – 12:00 Organizers meet to synthesize the workshop

- Workshop paper outline
  - Authorship
- Proposal ideas and outline
- Next steps
  - Exit survey

**Table S2** Shopping details

This supplement provides meal planning information to assist with the organization of workshops similar in size and participant preferences to the NEON GLUG kickoff workshop. It is intended as a starting point for others wishing to organize similar events. Key constraints relevant to the NEON GLUG workshop, which may differ in other situations, were as follows:

1.      Meals were planned for a 30-participant head count (maximum number of participants was 28). During workshop pre-planning, the distribution of participants identified the following dietary preferences and restrictions: meat-eater (22), vegetarian (6), vegan (3). None identified with gluten, peanut, or other food restrictions or allergies which made planning and execution of meals considerably easier. Other situations may differ.

2.      Workshop organizers made reservations for restaurants and carry-out food beginning one month in advance, with follow-up phone calls to establishments one week prior to the workshop for confirmation, and on the day of the meal in question. Other geographies may differ from the strongly seasonal, somewhat remote location of the NEON GLUG kickoff meeting, which was in northern Wisconsin after the primary summer tourist season.

3.      Workshop organizers conducted all shopping, which required nearly 3 hours, on the morning of the day before the workshop, at a large Wal-Mart. Other establishments may have similar capacities, though planners would do well to verify beforehand that they have access to a supermarket large enough to supply the considerable quantities needed to feed 25-30 people over a 2.5 day event. Organizers purchased ice and used large-capacity coolers to transport foods to the meeting venue.

4.      Cooking and dining facilities at the venue were fully equipped for the meals planned to occur at the venue.

The overall meal plan was as follows:

| **Day** | **Date** | **Meal** | **Menu** | **Item** |
| --- | --- | --- | --- | --- |
| W | 27-Sep | dinner | Carry out pasta, Wausau, WI | Selection of pasta |
| R | 28-Sep | breakfast | continental at Kemp | Bagel, toast, oatmeal, yogurt, fruit |
| R | 28-Sep | lunch | buffet at Kemp | Sandwich, salad, fruit, chips, cookies |
| R | 28-Sep | dinner | cookout @ Kemp | Burgers, kebabs, potato salad, pie |
| F | 29-Sep | breakfast | continental at Kemp | Bagel, toast, oatmeal, yogurt, fruit |
| F | 29-Sep | lunch | buffet at Kemp | Sandwich, salad, fruit, chips, cookies |
| F | 29-Sep | dinner | Minocqua Pizza Company | Private buffet |
| S | 30-Sep | breakfast | continental at Kemp | Bagel, toast, oatmeal, yogurt, fruit |
| S | 30-Sep | lunch | buffet at Kemp | Sandwich, salad, fruit, chips, cookies |

The specific geography enabled the pickup of hot and ready pasta dishes, in sealed foodservice pans, at about 16:00 local time on the afternoon before the workshop (Wednesday 27 September 2023). These dishes, which were planned in advance, were transported warm in coolers for the 1.5 hours of remaining road travel to the venue, and then served as a buffet to participants arriving the evening before the official start of the workshop.

One of the other meals (Dinner #2) was a reserved private buffet, planned in advance, at a pizzeria near the meeting venue.

All other meals were organized, prepared, served, and cleaned up by meeting organizers, with assistance from participants. Facilities enabled the use of safe food storage and hygiene practices. Future planners should consider important details such as refrigeration capacity, availability of cookware, flatware, utensils, and left-over food storage (and distribution) containers, in order to ensure that foods are safely and efficiently prepared, stored, and consumed or distributed.

Provided below is a detailed shopping list, with quantities, prices as of September 2023, and other notes, for workshops following a similar meal plan as the NEON GLUG kickoff meeting.

| **Meal** | **Category** | **Item** | **#pkgs** | **$/pkg** | **$ total** | **Notes** |
| --- | --- | --- | --- | --- | --- | --- |
| Breakfast * 3 | Bakery | Bagels | 6 | 3.75 | 23 | selection of kinds, 5 per pkg |
| Dinner 1 | Bakery | Buns | 2 | 3.78 | 8 | for venison burgers, 8 per pkg |
| Lunch * 3 | Bakery | Cookies | 10 | 3.98 | 40 | selection of kinds, 10 per pkg |
| Dinner 1 | Bakery | pies | 4 | 5.82 | 23 | 4 pies, fruit, assorted |
| Lunch * 3 | Bakery | Pita | 2 | 2.64 | 5 | 6 per pkg |
| Lunch * 3 | Bakery | Wheat bread | 10 | 4 | 40 | 10 loaves, assorted kinds |
| Breakfast * 3 | Dairy | Butter spread | 1 | 6.28 | 6 | 1 16oz tub |
| Breakfast * 3 | Dairy | Cream Cheese | 1 | 6.97 | 7 | 1 16oz tub |
| Snack/coffee | Dairy | half and half | 3 | 3.24 | 10 | 3 32 oz cartons |
| Breakfast * 3 | Dairy | Yogurt | 6 | 4.46 | 27 | 32 oz size |
| Lunch * 3 | Deli | sliced cheese |  |  | 50 | 3-4 pounds, various kinds |
| Dinner 1 | Deli | chicken breast | 1 | 15 | 15 | 1 5-6 pound tray |
| Dinner 1 | Deli | Ground Venison |  |  | 0 | 1 3-pound pkg ground venison (donated) |
| Lunch * 3 | Deli | Ham |  | 8 | 64 | 8 pounds, sliced |
| Lunch * 3 | Deli | Hummus | 2 | 4.97 | 10 | 17oz containers, 2 kinds |
| Multiple | Deli | sliced pickles | 2 | 4.66 | 9 | 2 16oz jars |
| Lunch * 3 | Deli | potato salad | 4 | 5 | 20 | 32 oz size |
| Lunch * 3 | Deli | Turkey |  |  | 63 | 6 pounds, sliced |
| Snack/coffee | Grocery | Mixed nuts | 2 | 14.98 | 30 | 30 oz container, for trail mix |
| Breakfast * 3 | Grocery | Fruit juices | 4 | 4 | 16 | orange, apple, cranberry |
| Dinner 1 | Grocery | baked beans | 2 | 2.5 | 5 | 2 28oz cans |
| Lunch * 3 | Grocery | Chips | 1 | 19.48 | 19 | 1 42-count variety box, misc chips |
| Snack/coffee | Grocery | Coffee | 2 | 13 | 26 | 2 30oz tubs |
| Snack/coffee | Grocery | Coffee filters | 1 | 5 | 5 |  |
| Dinner 1 | Grocery | cooking oil | 1 | 5 | 5 | 1 bottle |
| Snack/coffee | Grocery | Dried fruit | 2 | 6 | 12 | Craisins, blueberries |
| Multiple | Grocery | fizzy water | 9 | 8 | 72 | selection of flavors, 12per pkg |
| Breakfast * 3 | Grocery | Granola | 2 | 5.84 | 12 | 2 16oz bags |
| Snack/coffee | Grocery | Herbal tea | 3 | 3 | 9 | 3 boxes, different kinds |
| Multiple | Grocery | Honey | 1 | 11.98 | 12 |  |
| Dinner 1 | Grocery | Italian Dressing | 2 | 3 | 6 | for Dinner 1 kebab marinade |
| Breakfast * 3 | Grocery | Jam | 4 | 4.5 | 18 | several kinds |
| Dinner 1 | Grocery | Ketchup | 1 | 4.76 | 5 | 32 oz size |
| Dinner 1 | Grocery | spices |  |  | 10 | garlic powder, season salt, pepper |
| Dinner 1 | Grocery | Mayonnaise | 1 | 5.48 | 5 |  |
| Dinner 1 | Grocery | Mustard | 1 | 2.24 | 2 |  |
| Breakfast * 3 | Grocery | Instant oatmeal | 3 | 6.98 | 21 | 24 pouches per box, several flavors |
| Multiple | Grocery | Pbutter | 1 | 6.97 | 7 | 40 oz jar |
| Snack/coffee | Grocery | raisins | 1 | 4.72 | 5 | 20 oz, for trail mix |
| Dinner 1 | Grocery | red beans & rice | 2 | 2.74 | 5 | 2 boxes zatarain's |
| Multiple | Produce | Apples | 4 | 5 | 20 | 3# bags, several kinds |
| Multiple | Produce | Bananas | 2 | 2.25 | 5 | 2 bunches, 4lb ea |
| Multiple | Produce | Broccoli florets | 1 | 5.5 | 6 | ~2 pounds, for salads and snacks |
| Multiple | Produce | Carrot sticks | 6 | 2.44 | 15 | 12 oz per pkg, for salads and snacks |
| Multiple | Produce | Celery sticks | 6 | 2.98 | 18 | 12 oz per pkg, for salads and snacks |
| Multiple | Produce | cucumbers | 5 | 1 | 5 | for salads and snacks |
| Multiple | Produce | Salad greens | 6 | 4.98 | 30 | 16oz containers |
| Multiple | Produce | mushrooms | 4 | 2.28 | 9 | 8oz size, for salads & kebabs |
| Dinner 1 | Produce | onions | 2 | 1 | 2 | for kebabs |
| Multiple | Grocery | salad dressing | 4 | 4 | 16 | several kinds, incl. veggie dip |
| Multiple | Produce | oranges | 1 | 6.98 | 6.98 | 1 5lb bag clementines |
| Multiple | Produce | peppers | 6 | 2.98 | 18 | 3ct bags, for salads, snacks, kebabs |
| Multiple | Produce | Tomatoes | 4 | 2.28 | 9 | 10 oz pkgs, for salads and snacks |
| Dinner 1 | Produce | zucchini | 2 | 1.48 | 3 | 2 pounds, for kebabs |

**Table S3.** GLUG kick-off exit survey questions.

Thank you for participating in the NEON GLUG Kick-off Workshop. Please take some time to provide us with feedback so we can ensure our workshops cover relevant topics and so we can continue to improve our workshops for future participants. Please note that your answers will count as participation as a co-author in the subsequent workshop manuscript. All your responses will be anonymous.

| **Question** | **Options** |
| --- | --- |
| How did you learn about this workshop | Social media, NEON newsletter, TWG member, Colleague, Listserve, other. |
| What aspects of the Great Lakes User Group workshop went well? |  |
| What aspects of the Great Lakes User Group workshop could be improved? |  |
| What kinds of Great Lakes User Group workshop activities would you by interested in participating in, in future? | In-person GLUG workshop/conference, Virtual GLUG workshop/conference, Regular GLUG meetings (networking, updates, developing activities) |
| Please indicate your level of agreement with the following statements. Strongly disagree, Disagree, Neither agree nor disagree, Agree, Strongly agree. | The workshop was valuable to me  Attending the workshop was an overall good experience  I would recommend the workshop to my colleagues  I would attend future NEON workshops if offered |
| Do you have any other feedback for how to improve NEON workshops? |  |
| Please select your career stage | I do not wish to provide this information.  Undergraduate student  Graduate student  Early career (0-7 years post-terminal degree)  Mid-career (8-20 years post-terminal degree)  Early career (20+ years post-terminal degree) |
| What is your affiliation | Research university  Four-year college  Community college  Minority serving institution  Federal government  Local/state government  Public school (K-12)  Non-profit organization  Private sector  Other |
| Please select the discipline that best describes your domain of research, coursework or teaching. | Botany  Biogeochemistry  Conservation/restoration ecology  Ecosystem ecology  Education and outreach  Evolution/evolutionary ecology  Landscape ecology/biogeography  Microbiology  Physiological ecology  Population/community ecology  Wildlife biology  Zoology  Other |
| Please select your age range. | I do not wish to provide this information  18-24  25-34  35-44  45-54  55-64  65+ |
| Please select your gender | I do not wish to provide this information  Woman  Man  Non-binary  Other |
| Please select your race/ethnicity. Select all that apply. | I do not wish to provide this information  American Indian or Alaska Native  Asian  Black or African American  Hispanic or Latinx  Native Hawaiian or Pacific Islander  Middle Eastern  White  Other |
| Do you identify as a member of the LGBTQ+ community | I do not wish to provide this information  Yes  No |
| Please indicate if you have the following disabilities. Select all that apply. | I do not wish to provide this information  Hearing impairment  Visual impairment  Mobility/orthopedic impairment  None |
| Are you actively serving in the military or a military veteran | I do not wish to provide this information  Yes  No |

Thank you for taking time to provide us with your feedback. If you have any questions please contact us.
